# Low-Shrinkage 3D-Printed Polymeric Microneedle Arrays with Electroless Silver Coating for Enhanced Thermal and Mechanical Performance in Dermatological Applications

**DOI:** 10.3390/ma19183928

**Published:** 2026-09-16

**Authors:** Yafen Xu, Shuyan Li, Jun Liu, Zhongkai Li, Xiaofang Pan, Danya Li, Shoujing Mao, Wenxin Liu, Yangyang Li, Xin Guo, Yihong Tong, Gang Liu, Jieyue Wang

**Affiliations:** 1 School of Materials Science and Engineering, Central South University, Changsha 410083, China; 213102052@csu.edu.cn (Y.X.); 15211044693@163.com (S.L.); liujun4982004@csu.edu.cn (J.L.); lzk00595@163.com (Z.L.);; 2 Changsha Stomatological Hospital, Hunan University of Chinese Medicine, Changsha 410208, China; 3 Department of General Surgery, Xiangya Hospital, Central South University, Changsha 410083, China; 4 Eye Center of Xiangya Hospital, Central South University, Changsha 410008, China; 5 Hunan Key Laboratory of Ophthalmology, Central South University, Changsha 410083, China; 6 National Clinical Research Center for Geriatric Disorders, Xiangya Hospital, Central South University, Changsha 410008, China

**Keywords:** polymeric microneedles, additive manufacturing, low-shrinkage resin, electroless silver coating, energy-assisted dermatology

## Abstract

Microneedling has found various applications in dermatology recently. However, many problems still need to be solved in the fabrication of high-aspect-ratio polymeric microneedles with good mechanical strength, dimensional accuracy and thermal conductivity for energy-assisted treatments. Herein, a low-shrinkage photocurable resin specifically designed for high-precision additive manufacturing was developed in this work to enable the rapid and reproducible fabrication of ultra-long microneedle arrays through photopolymerisation-based 3D printing. Moreover, an even and uninterrupted layer of silver was added to the surface at room temperature via electroless plating. The resulting microneedle arrays have good structural integrity and high mechanical strength, and the thermal conductivity of them has been increased by a factor of 37 compared to that of uncoated polymeric materials. Mechanical tests showed that the compressive strength was high enough, and simulated skin and ex vivo porcine skin penetration experiments also demonstrated a low insertion force and stable microchannel formation. The in vitro cytotoxicity tests showed good cell compatibility both before and after silver coating. Such work has successfully developed an all-purpose and manufacturable microneedle platform that connects high-precision additive manufacturing with energy-based dermatological therapy, which provided a scalable strategy for expanding the application of polymeric microneedles in medical aesthetics and related clinical fields. The complete route from the digital model to the finished silver-coated array takes about 2.5 h and requires no mould, master or dedicated tooling. The arrays are intended to serve as low-cost, single-use and geometrically customisable needle-electrode cartridges for energy-assisted dermatological treatment and in particular for fractional radiofrequency (RF) microneedling.

## 1. Introduction

Skin-interface technologies for beauty and therapeutic purposes have drawn considerable attention in recent years, and the skin, as the largest organ of the human body and the principal barrier between the organism and its environment, has correspondingly become a major focus of this research. Among the above technologies, microneedle-based platforms have gradually emerged as minimally invasive, low-pain and highly effective ways and have been widely used in medical aesthetics for skin whitening, anti-ageing, acne treatment, etc.

Microneedles generally have arrays of tens to hundreds of micrometre-sized needle tips arranged in planar or roller-type formats. Microneedles have a small size and can be precisely controlled in terms of the depth at which they are inserted; thus, they can penetrate the stratum corneum to form temporary microchannels in the dermis without damaging the epidermis. Therefore, insertion is generally associated with minimal bleeding and pain because large blood vessels and nerve endings are not damaged [1]. These microchannels enhance transdermal transport significantly and can deliver both low-molecular-weight drugs as well as hydrophilic and high-molecular-weight molecules (>500 Da) to reduce the required drug dose [2,3,4,5,6,7,8,9,10,11]. Microneedle-mediated delivery avoids first-pass metabolism and is more suitable for drugs that degrade easily in the gastrointestinal environment compared with oral administration. Compared with subcutaneous injection, microneedles have lower pain and tissue damage; they are also less prone to pathogenic infection and easier for patients to perform themselves.

Microneedles can be used in conjunction with energy-type methods, such as light, electricity, and heat, to trigger multiple repair mechanisms in the epidermis and dermis; passively transporting molecules is also feasible. The combined effect of the two will result in better and longer regeneration, extending the application scope of microneedle-based therapy significantly. Among the above ways, microneedle-assisted radiofrequency (RF) therapy has been used most frequently. To stimulate the regeneration of collagen in the dermal layer by supplying thermal energy directly to this area, various types of skin rejuvenation [12,13,14,15,16,17] and dermatological treatments have been introduced; for instance, anti-acne medicine [18,19,20], scar therapy [21,22], treatment for enlarged pores, alopecia and alopecia areata [23,24], and melasma [25,26] all employ this approach. Compared with earlier trans-epidermal electrode devices, microneedle-assisted RF systems confine the electric field, and the Joule heating that it generates, to the needle–tissue interface inside the dermis; the epidermis is therefore largely bypassed, the energy-utilisation efficiency is increased and epidermal damage is reduced.

At present, stainless steel, titanium, ceramics and polymers are the materials generally used in the production of microneedles. Metallic microneedles are mainly made by electroplating, laser cutting and photochemical etching, and ceramic microneedles are generally prepared through micromoulding technology. Fabrication methods for polymeric microneedles that have been widely used include micromoulding, photolithography combined with micromoulding, injection moulding, hot embossing, lost-mould casting and drawing lithography, and these routes have recently been reviewed in detail. Although the above methods can produce microneedles with a certain degree of dimensional accuracy, they generally have a relatively complex processing process, require a master structure or a mould to be remade for every design iteration, have a high manufacturing cost, and have limited ability to produce intricate or personalised structures; thus, their continuous development and wide-ranging application have been restricted. As a result, the demand for high-precision structural control and functional optimisation manufacturing technology that is both cost-effective and scalable continues to rise.

Additive manufacturing is a comparatively recent fabrication route that offers a large design freedom and short production cycles at a low cost, and it is therefore particularly suitable for producing complex microstructures. In the present context, “design freedom” denotes the ability to build an essentially arbitrary three-dimensional geometry directly from a digital model, without any mould, master or dedicated tooling: features such as high aspect ratios, continuously varying tapers, sharp tips, undercuts and internal channels can be realised in a single build, and the needle length, taper angle, tip diameter, array pitch and needle number can be changed simply by editing the CAD file, so that a completely new array design is obtained within one print cycle. Recently, some promising results have been achieved in biomedical applications with 3D printing [27]. Among all 3D printing methods, photopolymerisation-based printing is relatively high-resolution and has good surface smoothness, so it is quite suitable for making microneedles. However, the material itself of photopolymerised polymeric microneedles has relatively poor thermal conductivity and is thus not well-suited for use in high-temperature radiofrequency-assisted microneedle therapy.

Based on the above problems, this paper has developed a 3D printing-enabled microneedle fabrication strategy that integrates material design, structural accuracy and functional surface modification. An ultra-low-shrinkage photocurable resin has been developed to achieve the rapid and reproducible fabrication of high-precision, high-strength, and ultra-long microneedle arrays through a simple photopolymerisation-based 3D printing process. A homogeneous and conformal thermally conductive layer can be produced on the surface of a microneedle using an uncomplicated electroless plating method, and thus significantly enhanced thermal conductivity can be achieved without an increase in insertion force. The above two methods can be used in combination to promote the expansion of applications for 3D-printed polymeric microneedles in medical aesthetics and other skin-interface therapeutic devices.

**Figure 1:**
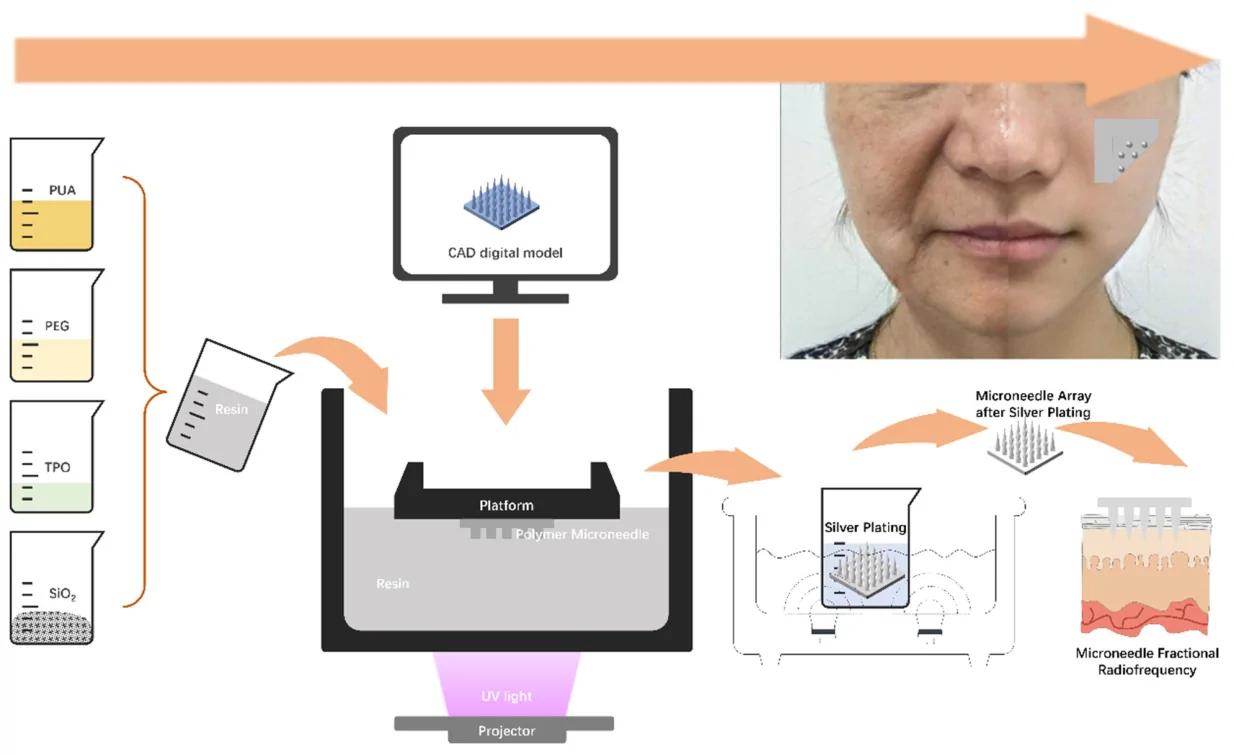
Schematic illustration of the fabrication process and working mechanism of the silver-coated 3D-printed polymeric microneedle array.

The intended biomedical application of the platform reported here is energy-assisted dermatological treatment and in particular fractional RF microneedling. In such treatments the needle array acts as the electrode that penetrates the stratum corneum and delivers RF energy, and the Joule heat generated by it, directly into the dermis, where controlled thermal zones trigger collagen remodelling; the clinical targets include skin rejuvenation and tightening, atrophic acne scars, enlarged pores, alopecia and melasma. The needle array in these devices is a single-use consumable, so the fabrication route has to be fast, inexpensive and easy to customise, while the needles must be long enough and strong enough to reach the target depth and thermally conductive enough to transfer the energy into the dermis instead of dissipating it inside the polymer body. The array developed here was therefore designed and characterised against exactly these requirements: dimensional fidelity of ultra-long needles (low-shrinkage resin), mechanical robustness and low insertion force (compression and penetration tests), efficient heat transfer (thermal conductivity), and biological safety (ISO 10993-5 cytotoxicity). Beyond RF microneedling, the same silver-coated arrays are of interest for other skin-interface devices that require electrical or thermal coupling to the dermis. It should be noted that the present study is confined to the materials level and to ex vivo validation; in vivo RF energy delivery, mapping of the resulting thermal field and histological evaluation of the thermal zones remain to be performed. Figure 1 illustrates the research concept and experimental process of this study.

## 2. Experimental Section

### 2.1. Preparation of High-Precision and High-Strength Photocurable Resin

All chemical reagents in this study were commercially obtained with analytical-grade purity or better and were not further purified. Ultrapure water (18.2 MΩ·cm), produced by a laboratory water-purification device, was used to make solutions and clean the samples.

A custom-prepared polyurethane acrylate (PUA, 30 g) and poly(ethylene glycol) diacrylate (PEGDA, 40 g) were weighed out and added to a 200 mL beaker. Homogenise the mixture using a magnetic stirrer at 300 rpm for 15 min. Next, 2 g of the photoinitiator 2,4,6-trimethylbenzoyldiphenyl phosphine oxide (TPO) was added to the mixture and then, at 60 °C, stirred for 30 min at 300 rpm to ensure that it was fully dissolved. The resulting solution was employed as the photocurable resin in the following 3D printing.

A PUA/PEGDA ratio of 3/4 was selected to balance the density of crosslinking and mechanical properties with reduced volumetric shrinkage, and thus good structural accuracy could be achieved in photopolymerisation-based 3D printing.

### 2.2. Design and 3D Printing of Microneedle Arrays

Computer-Aided Design (CAD) software (SolidWorks 2022) is employed to design the microneedle arrays. Each microneedle is a combined cylinder–cone type with a total length of 4500 μm; the cone tip is 3500 μm high, and the base of the cylinder is 1000 μm high. The lower-end diameter of all the microneedles was 750 µm. The size of the array was 10 mm × 10 mm, and it had a 5 × 5 arrangement.

A resolution of 8520 × 4320 was used by an LED-based 3D printer (ELEGOO, Shenzhen, China) to manufacture microneedle arrays. The printing parameters were established by a stepwise screening in which the layer thickness and the exposure time per layer were varied independently, and the resulting arrays were inspected by optical microscopy and SEM; the optimised values were a layer thickness of 25 μm and an exposure time of 8 s per layer, which correspond to 180 layers for a 4500 μm needle.

The layer thickness governs the Z-resolution and therefore the magnitude of the staircase effect on the conical surface of the needle. Thicker layers shorten the build time but generate visible steps along the taper, blunt the apex and lower the effective aspect ratio, whereas thinner layers increase the number of layers and hence both the build time and the number of separation events between the release film and the part, without a further gain in tip fidelity; 25 μm was the smallest layer thickness with which the 4500 μm needles could still be built reliably.

The exposure time per layer controls the cure depth and the extent of lateral light scattering into the surrounding resin. Insufficient exposure leaves the layer incompletely cured, which weakens interlayer adhesion and causes the slender tip to be lost during platform separation, whereas excessive exposure produces over-curing and light bleeding, which enlarges the XY dimensions, rounds the tip and increases the taper ratio. An exposure of 8 s per layer was the shortest time that gave fully cured, well-bonded layers together with sharp tips and no detectable dimensional growth, and it therefore represents the best compromise between dimensional accuracy, tip sharpness and mechanical strength of the microneedles.

Print them, then rinse the microneedle arrays with anhydrous ethanol to remove the surface of the uncured resin, and finally, post-cure under ultraviolet light (405 nm) in a UV curing chamber (UVGO) for 5 min.

## 3. Materials

Anhydrous ethanol, ammonium persulfate, ethylenediamine, stannous chloride, silver nitrate, ammonia solution, potassium hydroxide, anhydrous glucose and potassium sodium tartrate (all analytical grade) were purchased from Sinopharm Chemical Reagent Co., Ltd. (Beijing, China).

### 3.1. Electroless Plating Process

Add the polymeric microneedle arrays to anhydrous ethanol, place them in an ultrasonic cleaner at 30 °C for 10 min, and then remove the surface impurities. Then, the samples were added to a roughening solution containing ammonium persulfate ((NH_4_)_2_S_2_O_8_, 15 g/L) and sulfuric acid (100 mL/L) for 10 min under ultrasonic irradiation at 30 °C.

Then, the microneedles were sensitised by immersing them in a solution of concentrated hydrochloric acid (37%, 50 mL/L) and SnCl_2_·2H_2_O (30 g/L) for 20 min at 30 °C under ultrasonic agitation. Addition was made to a 10 g/L AgNO_3_ and a 30 mL/L NH_3_·H_2_O solution, and then, after stirring for 20 min at 30 °C under ultrasonic conditions, activation was carried out.

Electroless silver plating was performed by immersing the activated microneedles in a new plating solution at 30 °C for 10 min under ultrasonic vibration. The components of the plating solution were as follows: AgNO_3_ (14 g/L), NH_3_·H_2_O (60 mL/L), KOH (7 g/L), ethylenediamine (20 mL/L), glucose (8 g/L), potassium sodium tartrate (2.5 g/L), anhydrous ethanol (40 mL/L), polyethylene glycol (80 mg/L), and an adjustment to pH 13.0.

Ultrasonic agitation was used at all times during electroless plating to enhance the uniformity of mass transfer and uniform silver deposition on the high-aspect-ratio microneedle surface. The plating time was 10 min. This value was chosen because it was the shortest time that produced a continuous, pore-free silver layer over the whole needle, including the apex: with shorter times the deposit remained discontinuous and islanded near the tip, whereas longer times increased both the thickness and the roughness of the deposit and progressively blunted the fine tip. Under the conditions used here, 10 min of plating gave a coating thickness of 1.0–1.4 μm (Section 4.3) while the original tip geometry was preserved.

Plating was followed by a general-water rinse, and then an oven-drying method at 50 °C for 30 min was used.

### 3.2. Characterisation and Performance Evaluation

Fourier transform infrared (FTIR) spectra of the photocurable resin were acquired using an FTIR spectrometer (Nicolet iS50, Thermo Fisher Scientific, Waltham, MA, USA) in the range of 4000–500 cm^−1^ with a spectral resolution of 4 cm^−1^.

Based on ISO 3521:1997 [28], the volumetric shrinkage ratio of the resin was calculated as shown below:

$$\eta =\frac{v_{l}-v_{s}}{v_{l}}=1-\frac{\rho_{l}}{\rho_{s}}$$

where m is the mass of the resin, $\rho_{l}$ and $\rho_{s}$ are the densities of the liquid and solid resins, respectively, $v_{l}$ and $v_{s}$ are the corresponding volumes, and *η* is the resin shrinkage ratio. A smaller volumetric shrinkage rate is required to maintain the dimensions and structural integrity of the high-aspect-ratio microneedles after photopolymerisation.

A universal testing machine (HDW-20, Jinan, China) is available for the tensile tests at a speed of 0.2 mm/min in an environment that has been controlled. A 0.1 mm/min compression speed was used for the compression test at room temperature in the same device. All the mechanical tests were conducted on three or more independent samples, and the results were presented as mean ± standard deviation.

SEM was used to observe the shape and internal structure of the surface of the microneedle array (TESCAN MIRA4 LMH, Brno, Czech Republic).

Laser Flash Analysis (LFA 467, Netzsch, Selb, Germany) was used to measure thermal conductivity.

### 3.3. Skin Penetration Evaluation

The two supplementary means to be employed are presented below. A particular puncture-force test device was used to measure the insertion force of a single microneedle initially. A 3 wt% agarose gel was selected as the skin-mimicking substrate. Agarose hydrogels are among the most widely used skin simulants in microneedle insertion studies because they are transparent, homogeneous and reproducible and because their elastic modulus can be tuned through the polymer concentration to values comparable with those of human skin; at concentrations of about 2–3 wt% [29], the gels reproduce the resistance offered by skin closely enough to allow a quantitative and reproducible comparison of insertion forces between needle designs, and the same concentration has been used in previous penetration studies of 3D-printed microneedle arrays. A model substrate rather than real skin was used for this particular measurement because ex vivo skin varies considerably in thickness, hydration and mechanical properties, which makes a direct comparison of insertion forces between samples unreliable. At room temperature, a test was conducted at a penetration speed of 150 mm/min, and the maximum peak force achieved during this test was referred to as the insertion force.

Second, ex vivo porcine skin was employed for the penetration experiment because the structure, thickness and micromechanical behaviour of porcine skin closely resemble those of human skin. The porcine skin was commercially purchased from a local supermarket and originated from pigs slaughtered through the routine food supply chain. No animals were sacrificed specifically for this study, and no experimental procedures were performed on live animals [30]. Before adding it, the microneedle tips were stained with rose bengal B for better observation. Slowly press the microneedle array onto the skin of the pig for 10 s; then remove it. Ex vivo porcine skin was taken from a local abattoir, and no live animals were employed in this experiment. Immediately after withdrawal, a visual assessment of the skin surface and of the array itself was carried out under a stereomicroscope, and macroscopic images were recorded. The following criteria were checked: (i) the insertion success rate, defined as the number of stained puncture sites divided by the number of needles in the array (25); (ii) the spatial arrangement of the puncture sites, which had to reproduce the 5 × 5 pattern and the pitch of the array; (iii) the uniformity of the size and the shape of the individual dye spots; (iv) the absence of macroscopic skin damage such as tearing, laceration, dragging of the surface or bleeding around the puncture sites; and (v) the integrity of the microneedles after withdrawal, which were inspected for bending, blunting, fracture and loss of the silver coating. The same criteria were applied to the commercial microneedle array used for comparison. Then rinse the skin samples with distilled water, air-dry, fix in 10% neutral buffered formalin, gradually dehydrate with graded ethanol solutions, incubate in xylene, and embed in molten paraffin. A microtome was used to cut a 20 μm thick section of the embedded tissue, which was then placed on a glass slide. Deparaffinization and rehydration were carried out to stain the sections with haematoxylin and eosin (H&E), and an upright optical microscope was used for imaging of the microchannel.

### 3.4. Cytotoxicity Assay

Indirectly determine the toxicity of cells. Mouse fibroblast L929 cells (Chinese Academy of Sciences, Shanghai, China) were cultured in Dulbecco’s modified Eagle’s medium (DMEM) supplemented with 10% foetal bovine serum (FBS; Lonsera (Ciudad de la Costa, Uruguay)) and antibiotics (100 units/mL penicillin and 100 μg/mL streptomycin; Aladdin, Riverside, CA, USA) at 37 °C in a humidified atmosphere containing 5% CO_2_ for 72 h.

Microneedle samples before and after silver plating were sterilised with 100% isopropanol for 1 min and air-dried. According to ISO 10993-5:2009 [31], the samples were placed in culture medium at a surface area-to-volume ratio of 1.25 cm^2^/mL for 72 h to prepare the extraction medium. In line with ISO 10993-5, a collection concentration of 12.5 per unit mass was chosen for a sensitive and physiologically meaningful assay of cytotoxicity.

Dilute the extracts in fresh culture medium and then use them to culture L929 cells for 1, 3 and 5 days. After the specified incubation time, 10 μL of CCK-8 solution (Dojindo, Kumamoto, Japan) was added to each well and then incubated for 2 h. Biotech GEN5 Microplate Reader (BioTek Instruments, Inc., Winooski, VT, USA) is employed for the 450 nm absorbance measurement. The formula for cell viability [31] is as follows:

$$\mathrm{Cell}\;\mathrm{viability}\;(\%)=\frac{OD_{t}-OD_{blank}}{OD_{nc}-OD_{blank}}\times 100\%$$

*OD_t_* is the absorbance of the experimental group, OD*_nc_* is the absorbance of the negative control group, and *OD_blank_* is the absorbance of the DMEM.

Live-cell staining was also used to study cytotoxicity of L929 cells in the extraction medium at 1, 3, and 5 days. Axio Observer 5 digital inverted fluorescence microscopy (Carl Zeiss, Thornwood, NY, USA) was used to view live cells exhibiting green fluorescence.

## 4. Results and Discussion

### 4.1. Spectral Analysis of the High-Precision Photocurable Resin

Photopolymerisation-based 3D printing has achieved good accuracy and repeatability in the size, shape and distance among all microneedles in an array, which is a prerequisite for a uniform insertion behaviour across the whole array. Polyethylene glycol diacrylate (PEGDA) is a good, biocompatible material with high mechanical strength, and it has already been shown to be printable into high-aspect-ratio microneedles by photopolymerisation, so it has been selected as the matrix material for microneedle construction. PEGDA is a hydrogel-based material with good biocompatibility and tunable mechanical properties (such as strength and flexibility) and adjustable curing kinetics; thus, it is highly suitable for a large number of biomedical applications, including 3D bioprinting of tissues [32,33], drug delivery systems, and cell culture scaffolds [34,35].

Polyurethane acrylate (PUA) has strong polarity of the –NH–CO–NH– structure in its molecular chain and is thus relatively strong and hard [36]. Therefore, PUA was added to the PEGDA matrix to increase the strength and toughness of the microneedles. Design of the material has reduced the force required to penetrate the skin and thus minimised pain; at the same time, it can still meet the structural requirements under uneven loading due to angular misalignment during insertion into elastic skin tissue.

**Figure 2:**
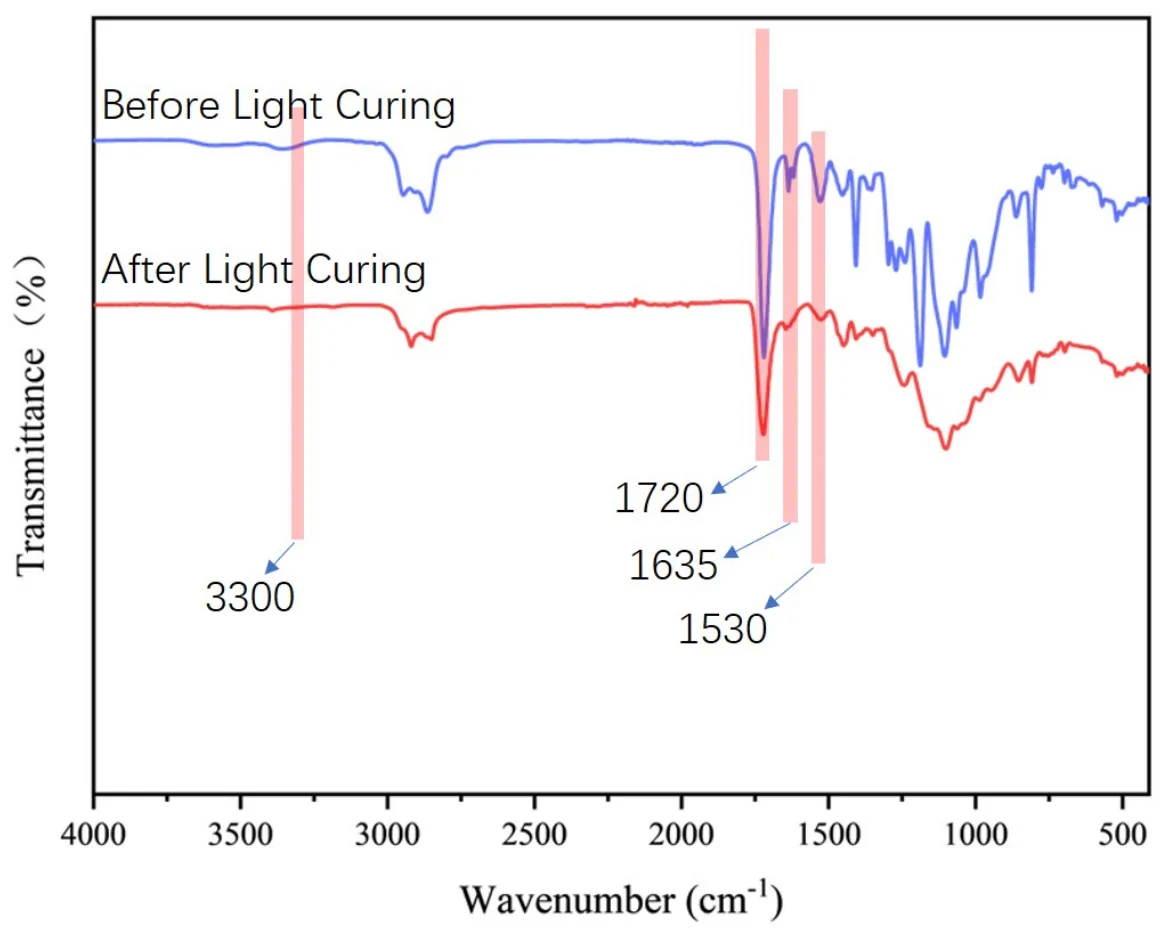
FTIR spectra of the photocurable resin before and after photopolymerization.

The Fourier transform infrared (FTIR) spectra of the photocurable resin before and after photopolymerisation are shown in Figure 2. After curing, the characteristic absorption peak around 1635 cm^−1^, corresponding to the C=C double bonds of PEGDA and PUA, was significantly reduced or disappeared; thus, a high degree of conversion through free-radical polymerization and the formation of a crosslinked network had been achieved. The absorption bands around 3300 cm^−1^ and 1530 cm^−1^ showed no shift in position or change in intensity; thus, the urethane –NH–CO–O– groups did not participate in the photopolymerization reaction. The C=O stretching vibrations of PEGDA and PUA at approximately 1720 cm^−1^ exhibited minor changes in peak shape; this is likely due to a modification of the local chemical environment after crosslinking, but no new peaks were generated.

### 4.2. Volumetric Shrinkage of the Photocurable Resin

Volumetric shrinkage in photopolymerisation is caused by the conversion of carbon–carbon double bonds into single bonds, resulting in a reduction in free volume in the cured material [37,38]. Photopolymerisation-based 3D printing is done in layers, and as such, volumetric shrinkage occurs over time during the printing process. Too much shrinkage can result in poor interlayer adhesion [39], microcracks or voids [40], residual stress [41], and large-scale deformation such as curling or warping [42].

Therefore, the reduction in volumetric shrinkage needs to be achieved to improve the dimensional accuracy of photopolymerisation-based 3D printing. A highly photocurable resin developed in this study shows a very low volumetric shrinkage of 1.81 ± 0.14%. A comparison with other reported resin systems is shown in Table 1, and the proposed formulation has exhibited better dimensional stability and is suitable for fabricating high-aspect-ratio microneedle structures.

### 4.3. Morphology and Structure of the Microneedle Arrays

**Figure 3:**
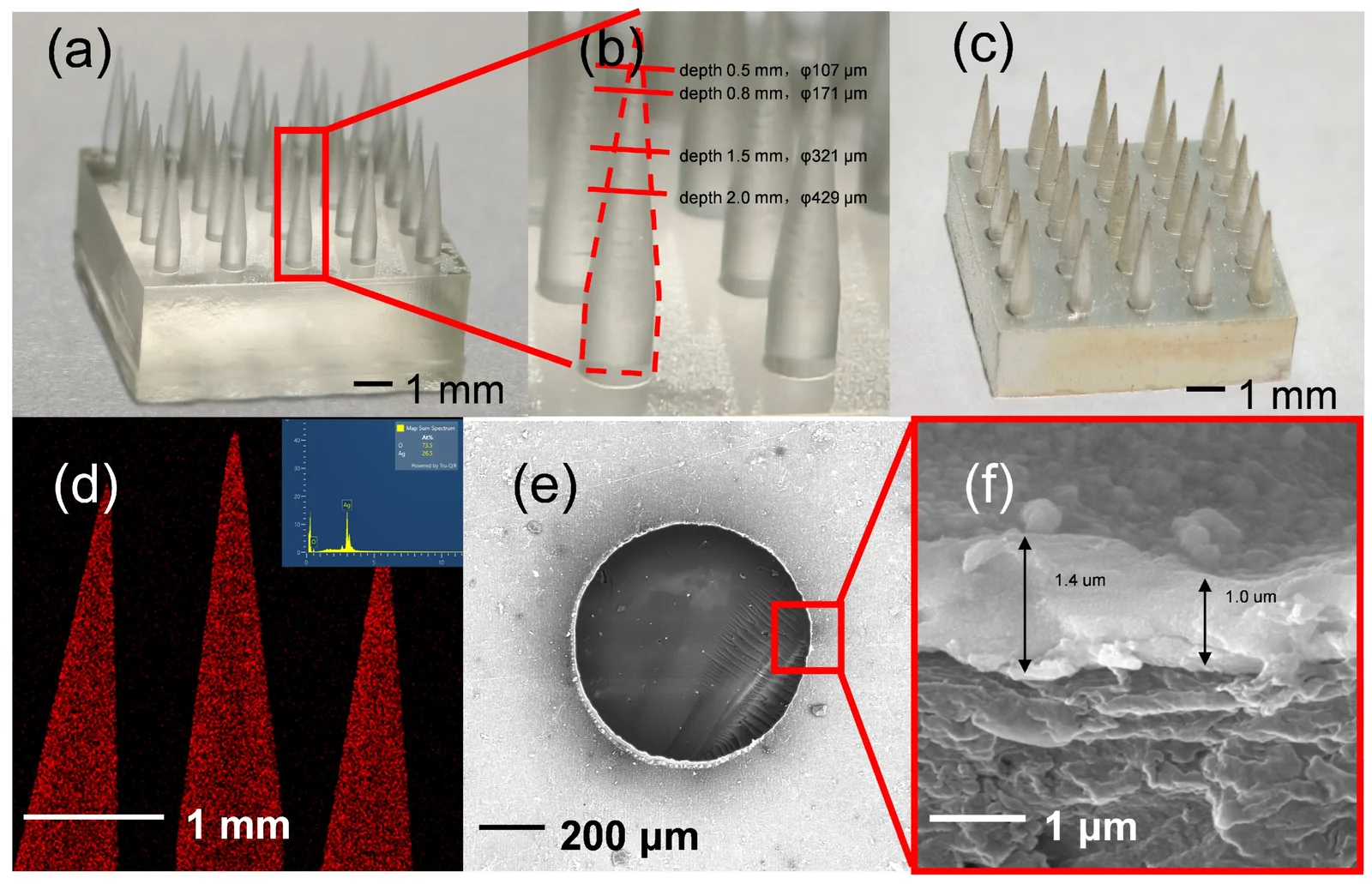
Morphology and microstructural characterisation of polymer microneedle arrays before and after electroless silver plating. (**a**) Optical image of the as-printed polymer microneedle array before silver plating. (**b**) Diameter variation of a single microneedle at different axial positions from the needle tip. (**c**) Optical image of the microneedle array after electroless silver plating. (**d**) Energy-dispersive X-ray spectroscopy (EDS) elemental map of the silver-plated microneedles, showing a uniform distribution of Ag. (**e**) Cross-sectional SEM image of a silver-plated microneedle showing the coating morphology. (**f**) High-magnification SEM image of the microneedle cross-section, highlighting the thickness of the electroless silver coating.

Microneedles loaded with drugs generally require a larger surface area and are often square pyramids or grooved structures [48]; in contrast, the microneedles created in this study aim to be gently inserted and withdrawn from the skin. To reduce damage and friction of the tissue, the shafts of the microneedles were designed to be cylindrical and conical in shape, and they had a smooth surface. Surface smoothness was evaluated qualitatively from the SEM images of Figure 3c,e,f, which were recorded at magnifications between ×100 and ×5000 along the shaft and at the apex: no layer-induced staircase steps, delamination, cracks, pores or adhering uncured resin could be resolved on the needle surface, and the transition from the cylindrical base to the conical tip was continuous. The same images were used to confirm that the electroless silver layer replicated this surface without nodular growth. A quantitative determination of the surface roughness of the curved needle surface (for example, the arithmetic mean roughness Ra by laser confocal microscopy or white-light interferometry) was not carried out in the present work and is planned for the follow-up study.

Figure 3a is a 5 × 5 microneedle array made by 3D printing, and it has good shape accuracy. The total height of each microneedle was 4500 μm; the height of the conical tip was 3500 μm; the base diameter was 750 μm; the taper ratio was about 0.21; and the tip angle was around 12.23°. Notably, the fabricated microneedles had an ultrahigh aspect ratio and were significantly larger than those of conventional polymeric microneedles, typically only 10–2000 μm in height [49,50,51,52].

A comment on terminology is appropriate at this point. Although the total length of the needles described here lies in the millimetre range, the structures are still referred to as a microneedle array because in this field the prefix “micro” refers to the micrometre-scale cross-section of the individual needles and to the micrometre-scale channels that they create in the skin, not to the overall needle length. The needles reported here have a tip angle of 12.23° and a shaft diameter of only 107 μm at the position closest to the apex (Figure 3b), so that the parts of the structure that interact with the tissue, and the microchannels formed in it, are unambiguously micrometre-sized. Needle lengths of this order are also standard in the microneedle literature and in commercial microneedling devices: drawing lithography has been used to produce ultrahigh-aspect-ratio microneedles of comparable length, and clinical RF microneedling systems are routinely operated at penetration depths of 0.5–3.5 mm. Where the distinction matters, the present structures may be described more precisely as ultra-long, ultrahigh-aspect-ratio microneedles.

Although ultrahigh-aspect-ratio microneedles are often associated with problems of mechanical instability and fracture during insertion, the high compressive strength of PUA-modified resin has reduced this risk, as shown in the following mechanical and penetration tests.

The clinical effects of different penetration depths in radiofrequency (RF) microneedle therapy vary accordingly; for instance, around 0.5 mm is used for skin rejuvenation; approximately 0.8 mm is selected to reduce pores and tighten the skin; about 1.5 mm and 2.0 mm are applied for wrinkle reduction and lifting; and at depths of up to 2.0 mm, deep scars can also be treated [53]. However, due to the soft, elastic and non-planar characteristics of skin, considerable bending and deformation occur during insertion, and the actual penetration depth is significantly shallower than the length of the microneedle (e.g., a microneedle with a total length of 900 μm may only penetrate about 300 μm) [54].

To prevent damage to the skin and ensure that the actual depth of penetration at the treatment site can be realised, a relatively long microneedle must be employed. Therefore, the length used in this experiment was 4.5 mm. As shown in Figure 3b, the diameters of the microneedles at some functional lengths were 107 μm, 171 μm, 321 μm and 429 μm, respectively.

Compared with the traditional polymer microneedle manufacturing processes of etching, micromolding, photopolymerization reaction and drawing lithography, the 3D printing method for developed photocurable resins has been selected due to its quick prototyping, simple processing, excellent shape uniformity and consistent sharpness of the tip. Together, the developed photocurable resin and additive manufacturing strategy can achieve precise control over the geometry of microneedles and maintain good structural uniformity and a sharp tip; this is difficult to achieve simultaneously with traditional polymeric microneedle fabrication methods. As an additive manufacturing technique, it is relatively environmentally friendly and has reduced material waste compared with subtractive processes.

To substantiate the claim of “quick prototyping, simple processing”, the time required by each step of the present route was recorded. Once the CAD model is available, printing one 5 × 5 array (180 layers of 25 μm at 8 s exposure per layer) takes about 30 min including the platform movements between layers. Because the whole 8520 × 4320 build area is exposed simultaneously, the printing time is essentially independent of the number of arrays placed on the platform, so that several tens of the 10 mm × 10 mm arrays used here can be produced within the same 30 min. Rinsing in anhydrous ethanol and UV post-curing add 5 min each. The electroless plating sequence takes about 100 min in total (ultrasonic cleaning 10 min, roughening 10 min, sensitisation 20 min, activation 20 min, plating 10 min, rinsing and drying 30 min) and is likewise a batch process. The complete route from digital model to finished silver-coated array therefore takes approximately 2.5 h, of which less than 45 min are needed for the polymeric array itself, and no mould, master or dedicated tooling is required at any stage.

For comparison, the conventional routes summarised in Table 2 all require a master structure or a mould to be produced before the first needle can be replicated. Micromoulding and photolithography-based routes need a silicon or SU-8 master fabricated by lithography and etching in a cleanroom, which typically takes from one day to several days and has to be repeated whenever the geometry is changed; injection moulding and hot embossing require a metal tool whose manufacture takes weeks and is orders of magnitude more expensive, so that these routes are economical only at large production volumes; and drawing lithography yields ultrahigh-aspect-ratio needles but relies on a sequential, temperature-controlled drawing step for every array. In the present work a change in geometry requires only the CAD file to be edited, and the modified array is available within the same working day, which is the main practical advantage of the proposed strategy for a device that is disposable and, potentially, patient-specific.

Electroless silver plating will be used for better stability and heat transfer. Silver has good thermal conductivity (429 W·m^−1^·K^−1^ at room temperature; it is higher than that of gold at 317 W·m^−1^·K^−1^), is broad-spectrum antibacterial without easily inducing resistance [55,56], and is thus widely applied in biomedical fields. Electroless silver plating of medical polymers offers a cost-effective, simple and non-directional option to ion-beam-assisted deposition methods that typically require complex vacuum systems and line-of-sight processing [57]. No special equipment was available for a low-temperature plating process in this study.

In addition to the above, an uninterrupted silver layer can also enhance electrical coupling and is therefore suitable for energy-based microneedle treatment, such as radiofrequency therapy. Previous research has reported adhesion strengths of electroless silver coatings in the range of 8.5–12 N, which are sufficient to withstand the insertion force of the skin [58,59]. Although the strong acid- or base-pretreatment before plating may damage or blunt the tips of microneedles [60,61,62], the pretreatment conditions in this study were strictly controlled.

As shown in Figure 3a, the polymeric microneedles were transparent before plating and had a uniform matte silver colour after electroless deposition. Importantly, the silver-coated microneedles retained a sharp-tip geometry (Figure 3c), and thus the pretreatment had not damaged the tip. A very thin, uniform layer of silver is added to the top of the microneedles to improve thermal conductivity and prevent swelling of the polymer material caused by moisture absorption.

EDS mapping is shown in Figure 3d to demonstrate that silver is uniformly distributed on the surface of microneedles; separate silver peaks also confirm the chemical purity of the coating. Cross-sectional SEM images (Figure 3e) show that there is a continuous and uniform silver layer with a thickness of 1.0–1.4 μm (Figure 3f); such a thickness is sufficient to improve the thermal performance of polymeric microneedles significantly.

### 4.4. Thermal and Mechanical Properties

**Figure 4:**
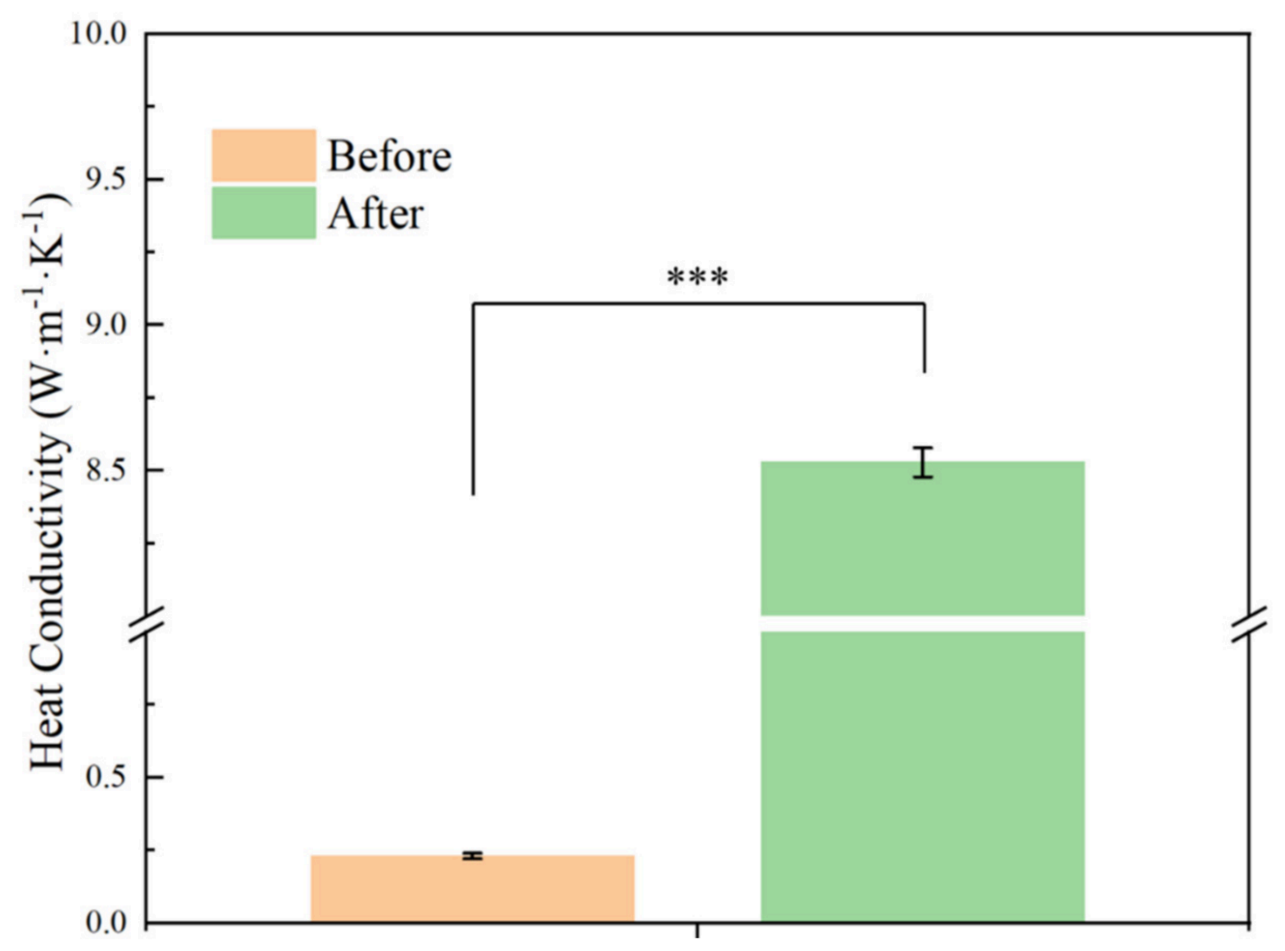
Thermal conductivity of polymer microneedle arrays before and after electroless silver plating (*** represents statistical difference, *p* < 0.001, n = 3).

As shown in Figure 4, the thermal conductivity of the uncoated polymeric microneedle array was only 0.23 W·m^−1^·K^−1^. Electroless silver plating dramatically increased the thermal conductivity to 8.53 W·m^−1^·K^−1^ (316 stainless steel: 14.5–15.2 W·m^−1^·K^−1^), an increase of about 37 times. The main reasons for the significant improvement are that silver has high intrinsic thermal conductivity and can be uniformly coated on the surface of the microneedles. The thermal conductivity at this level will reduce the amount of heat dissipation at the microneedle-tissue interface significantly and thus ensure that the energy delivery in RF-assisted microneedle therapy is both effective and localised.

**Figure 5:**
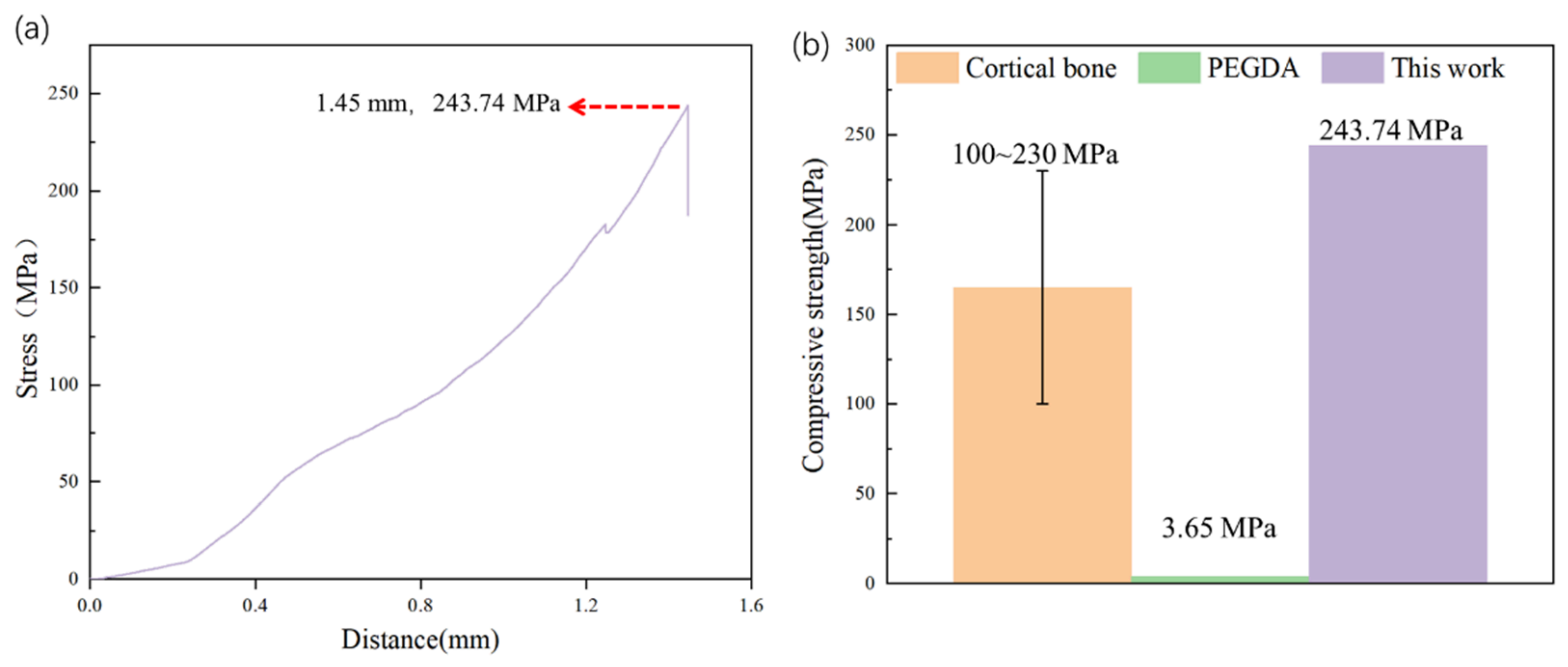
Compressive mechanical performance of the photocurable resin after electroless silver plating. (**a**) Compressive stress-displacement curve of the resin developed in this study. (**b**) Comparison of compressive strength between cortical bone (reported range), PEGDA-based resin with low strength (reported value: 3.65 MPa), and the resin developed in this study (243.74 MPa).

The compressive strength of microneedles made from pure PEGDA resin by 3D printing has been reported as only 3.65 MPa [63]. Generally speaking, the two typical ways to improve the mechanical properties are to blend with other resins or to add fibres, fillers and nanoparticles. PUA was added to increase the impact strength of the photocurable resin in this experiment. As shown in Figure 5, the compressive strength was 243.74 MPa; that of pure PEGDA was about 61 times lower, and it was roughly the same as that of cortical bone (100–230 MPa). It has relatively large mechanical strength and can penetrate the stratum corneum of the skin repeatedly.

**Figure 6:**
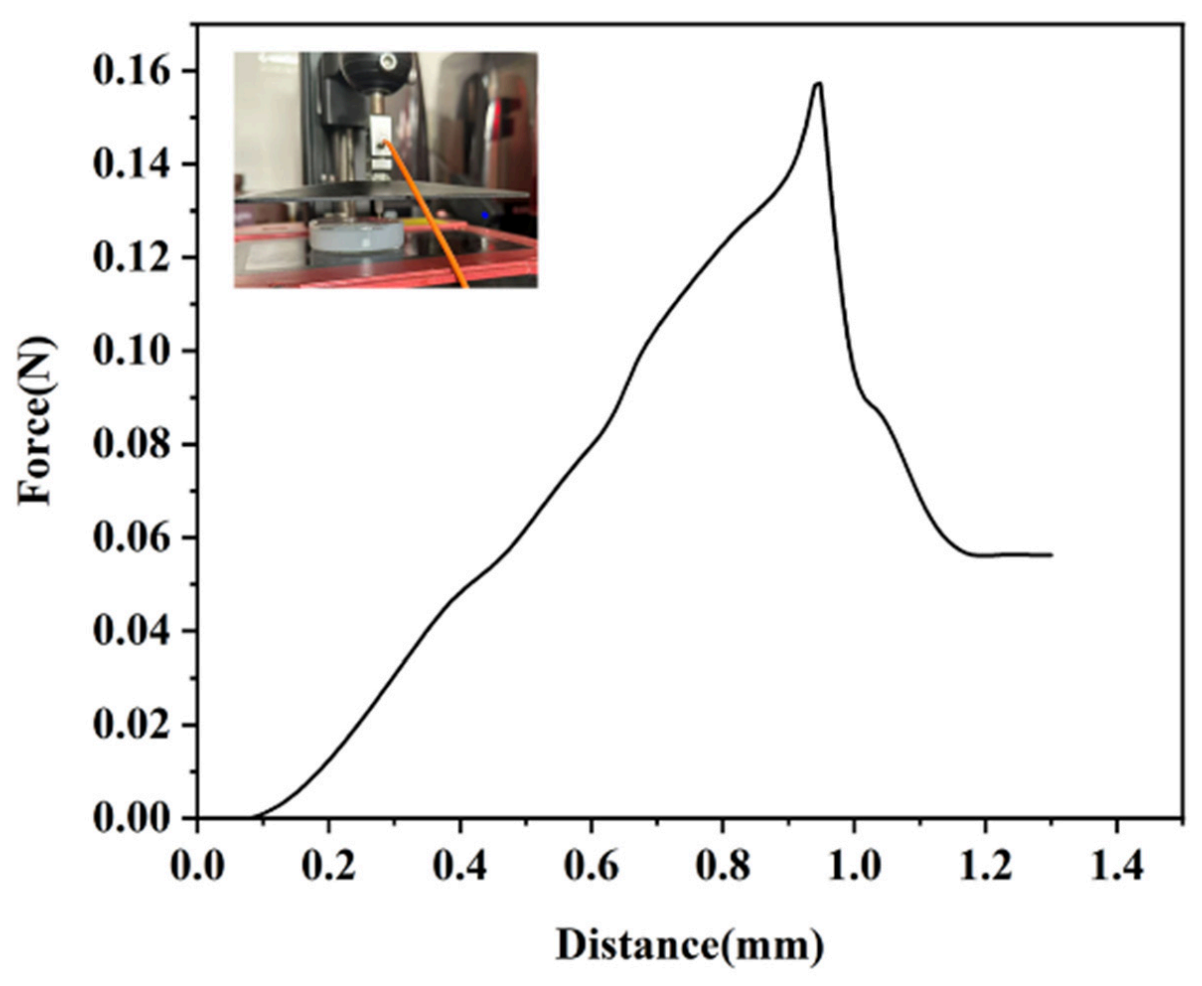
Penetration force of the polymer microneedle after electroless silver plating.

Further test the penetration performance of skin using a simulated skin-insertion device and ex vivo animal experiments. Figure 6 shows that in the skin simulation test based on agarose gel, the measured insertion force was only 0.15 N, which is approximately one-tenth or even less of the insertion force of polymer microneedles reported in the other literature (typically ranging from 1 to 7.3 N) [29,62].

**Figure 7:**
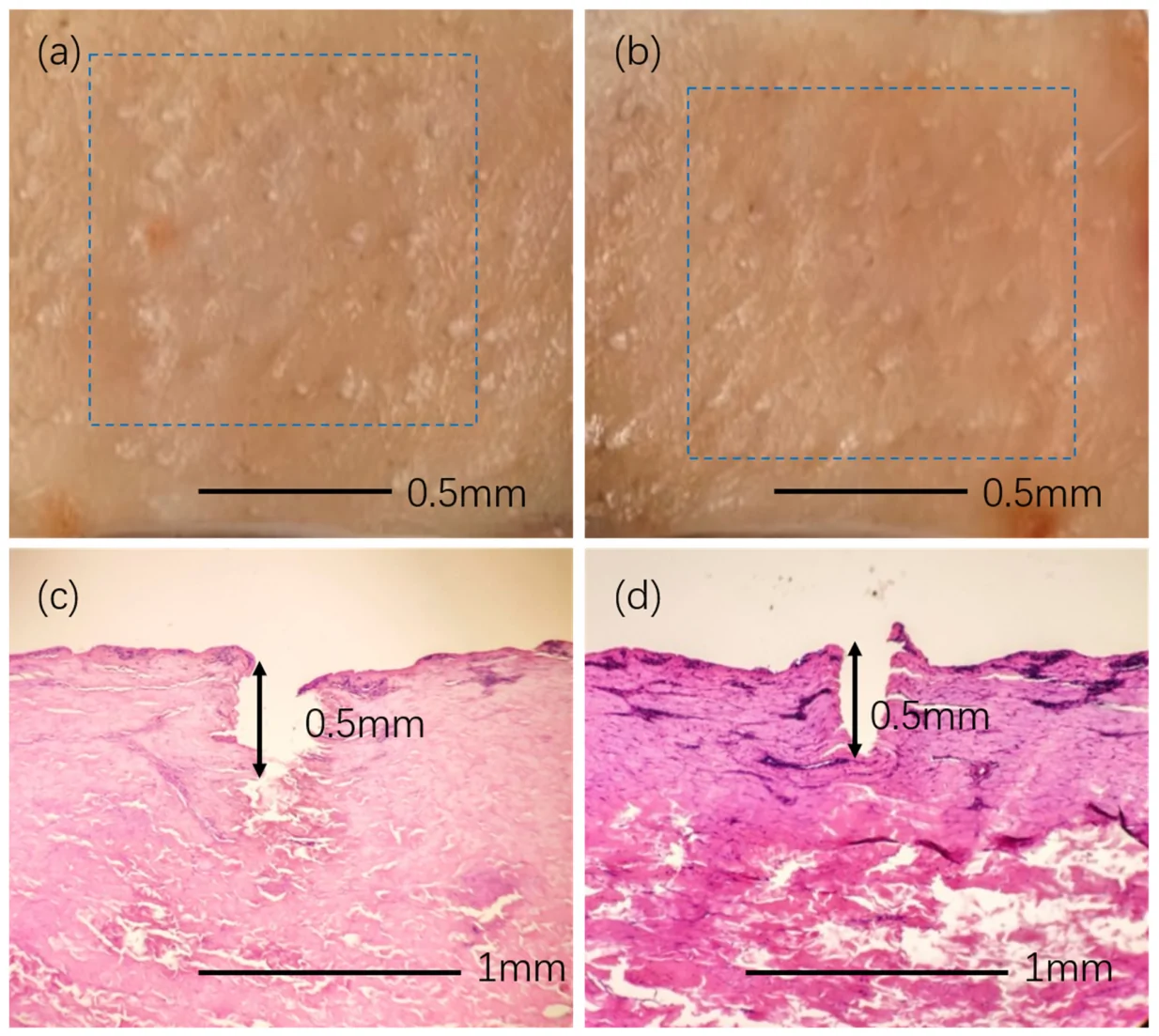
Evaluation of transdermal penetration performance of two microneedle types. (**a**,**b**) Macroscopic images of porcine skin after penetration by microneedle A and microneedle B, respectively (scale bar: 0.5 mm). (**c**,**d**) Corresponding H&E-stained histological sections showing the penetration depth and microchannel formation induced by the microneedles (scale bar: 1 mm).

Due to the similar structure of porcine and human skin, porcine skin was chosen for the subsequent experiments. Figure 7a,b show that both the polymeric microneedles and the commercial microneedles produced uniformly distributed and similarly sized puncture sites on the surface of porcine skin, and there was no visible damage.

Haematoxylin and eosin (H&E) staining images (Figure 7c,d) also show that both the polymeric and commercial microneedle arrays have successfully penetrated the stratum corneum to form microchannels with a depth of approximately 500 μm. Collectively, an ultra-low-shrinkage photocurable resin, an ultrahigh-aspect-ratio microneedle geometry and a conformal silver coating have been established with clear structure–property–function relationships to achieve a single-polymer microneedle platform with low insertion force, high mechanical robustness and efficient thermal transport.

The commercial device used for comparison (microneedle B in Figure 7) is a commercial microneedling cartridge (Derma Shine, Seongnam-si, Korea), containing 25 needles in a square matrix array; the needles are made of medical metal, are almost 5 mm long with a base diameter of 0.23 mm, and the recommended penetration depth of the device is 0.1–3.5 mm. It was applied to the porcine skin under exactly the same conditions as the array reported here, i.e., by the same operator, with the same applied force and dwell time, and with the same staining, fixation and sectioning protocol.

Under these conditions the two arrays performed comparably: both produced regularly distributed puncture sites of similar size without visible surface damage (Figure 7a,b), and both formed microchannels of approximately 500 μm depth in the histological sections (Figure 7c,d). The purpose of the comparison is therefore not to show that the polymeric array penetrates better than the metallic one, but that it reaches the performance of an established metallic device by a fundamentally simpler and far more flexible route. The advantages of the present system follow from this equivalence. First, the array is produced directly from a CAD file without any mould or tooling, so that needle length, taper, pitch and number can be adapted to the indication, or to the individual patient, within a single print cycle, whereas the geometry of a metallic cartridge is fixed by its tooling. Second, the cost per array is low and the process is batch-parallel (Table 2), which suits a single-use consumable. Third, the measured insertion force per needle is only 0.15 N, well below the 1–7.3 N reported for polymeric microneedles, which is relevant for patient comfort. Fourth, the electroless silver layer raises the thermal conductivity to 8.53 W·m^−1^·K^−1^, the same order of magnitude as 316 stainless steel (14.5–15.2 W·m^−1^·K^−1^), and therefore brings a polymeric needle into the range required for energy-assisted treatment while retaining the low density, low cost and design freedom of a polymer. It should nevertheless be stated explicitly that the metallic device still has the higher bulk thermal conductivity and that a head-to-head comparison of the RF energy actually delivered in vivo has not yet been performed.

### 4.5. Cytotoxicity Evaluation

**Figure 8:**
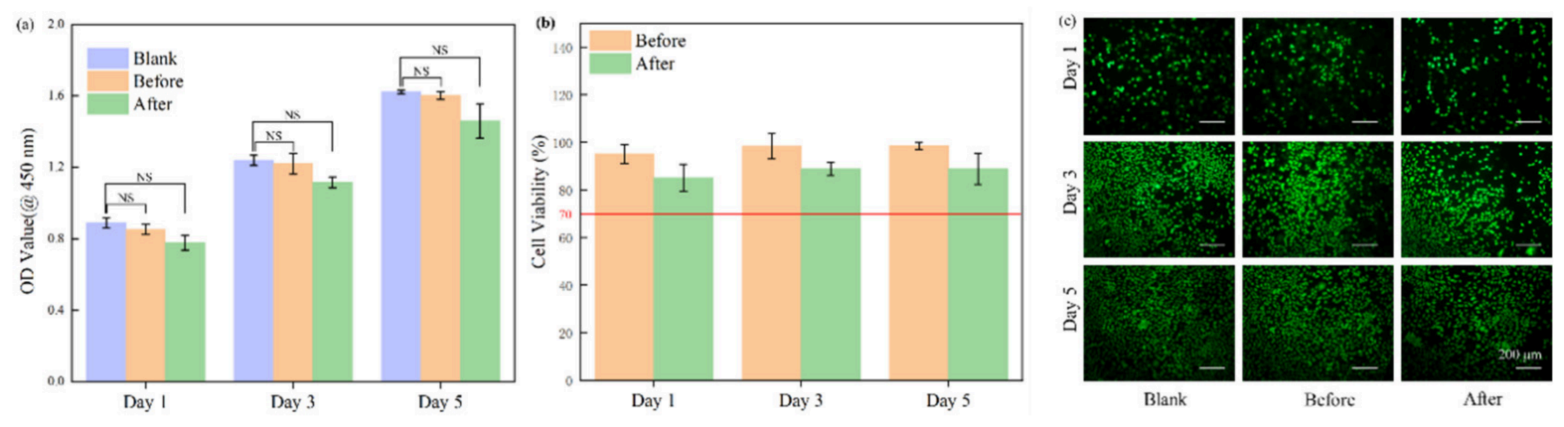
In vitro cytocompatibility evaluation of microneedle arrays. (**a**) CCK-8 assay absorbance (OD values) of L929 cells cultured with microneedle extracts for 1, 3, and 5 days (NS: not statistically significant compared with the blank control). (**b**) Cell viability of L929 cells after exposure to microneedle extracts for 1, 3, and 5 days. (**c**) Live/dead fluorescence staining of L929 cells cultured in microneedle extracts for 1, 3, and 5 days. (NS represents no statistical difference, *p* > 0.05, n = 3).

Biocompatibility of the microneedle arrays is needed to ensure the safety of their use in biomedicine. The L929 mouse fibroblast cell line is commonly employed to test the biocompatibility of the new material [64]. As shown in Figure 8, at days 1, 3 and 5, the extracts from microneedles before and after silver plating did not significantly differ from the blank control group in L929 cell viability.

Uncoated samples were no better than the silver-coated microneedles for reducing cell viability; all samples still showed a cell viability of more than 80%, well above the general threshold of 70%. In addition, live-cell staining images showed that all the groups had a similar number of live cells and normal cell shapes and growth. Based on the above results, it is found that there is a good chance for further in vivo studies, and the silver-coated polymeric microneedle platform may have some clinical application in dermatology.

## 5. Conclusions

A low-shrinkage photocurable resin specifically designed to address the problems of dimensional distortion and mechanical instability in high-aspect-ratio microneedles was successfully developed in this study. By combining high-resolution photopolymerisation-based 3D printing with a room-temperature electroless silver plating process, polymeric microneedle arrays with good structural fidelity were rapidly produced.

The resulting microneedle arrays had good mechanical strength and significantly improved thermal performance. Electroless silver plating increased the thermal conductivity of the uncoated polymeric microneedles by 37 times and is thus more suitable for effective heat transfer in radiofrequency-assisted skin treatment. Based on the above mechanical and penetration tests, it has been confirmed that the microneedles are sufficiently strong to repeatedly pierce the stratum corneum with a small insertion force, create stable microchannels, and cause only minimal damage to the tissue.

In addition, according to ISO 10993-5, in vitro cytotoxicity tests were performed to confirm that there was no harm to cells before and after silver coating, and thus the microneedle array could be used in biomedicine.

The intended application of this platform is energy-assisted dermatological therapy, and in particular fractional RF microneedling, in which the array serves as a low-cost, single-use and geometrically customisable needle-electrode cartridge that conducts heat into the dermis. The complete route from CAD model to finished silver-coated array takes about 2.5 h and requires no mould or dedicated tooling, so that a new geometry can be realised within a single working day. The main limitation of the present study is that the validation is confined to the materials level and to ex vivo tests: in vivo RF energy delivery, mapping of the resulting thermal zones, histological assessment of the thermal damage, the durability of the silver layer over repeated insertions and the release of silver ions over a clinically relevant period remain to be evaluated, and these aspects are the subject of ongoing work.

In short, this paper presents an all-purpose and easily manufactured microneedle platform that integrates high-precision additive manufacturing with energy-based dermatological therapy to provide a scalable path for expanding the application scope of polymer-based microneedles in medical aesthetics and other clinical fields.

## Figures and Tables

**Table 1:** Comparison of resin compositions, processing methods, and volumetric shrinkage of reported photopolymer resins for 3D printing.

Article Source (Year)	Resin Composition	Processing Method	Volumetric Shrinkage (%)
Deng et al. [43]	Urethane Acrylate (RJ 426), Monofunctional Diluents (RJ 425, IBMA), Crosslinker (TMPTA)	DLP 3D Printing Layer Thickness: 50 μm	4.15 ± 0.26% (A2 Formulation)
Zhao et al. [44]	Bis-GMA/TEGDMA, Silanized micro/nano-SiO_2_	DIW 3D Printing Layer Thickness: 410 μm	2.58 ± 0.11% (3D-P25M59N16)
Li et al. [45]	Bisphenol A Epoxy Acrylate (EA), DPGDA, Thiol-terminated Hyperbranched Polymer (T-HBP)	DLP 3D Printing Layer Thickness: 0.05 mm	Reduced by ~45.5% (with 20 wt% T-HBP)
Li et al. [46]	Bio-oil-modified Polyurethane Acrylate (PUA), PEG400DA	LCD 3D Printing Layer Thickness: 0.05 mm	2.65 ± 0.19% (20BPUA)
Pruksawan et al. [47]	HDDA, Carbon Black/Pigment, TPO	DLP 3D Printing Layer Thickness: 0.05 mm	13.5 ± 0.67% (Base HDDA)
This work	Polyurethane Acrylate (PUA), PEG200DA, SiO_2_, TPO	LCD 3D Printing Layer Thickness: 25 μm	1.81 ± 0.14%

**Table 2:** Comparison of the fabrication route developed in this work with conventional routes for polymeric microneedle arrays.

Fabrication Route	Master/Mould/Tooling Required	Lead Time for the First Array of a New Geometry	Cycle Time per Array (Batch Production)	Geometry Change
Micromoulding [31]	Yes (master + PDMS mould)	1–several days (cleanroom lithography/etching)	Tens of minutes (casting + demoulding)	New master and mould required
Photolithography + micromoulding [32]	Yes (SU-8 master + mould)	Several days	Tens of minutes	New master and mould required
Injection moulding/hot embossing	Yes (metal tool)	Weeks; high tooling cost	Seconds–minutes (economical only at high volume)	New metal tool required
Drawing lithography [33]	No mould, but sequential drawing	Hours	Sequential, one array at a time	Process parameters must be re-tuned
3D printing of the low-shrinkage resin + electroless silver plating (this work)	None	About 2.5 h from CAD model to finished silver-coated array	About 30 min for the whole build platform (several tens of arrays in parallel) plus 100 min batch plating	Edit the CAD file only; new array within the same working day

## Data Availability

The original contributions presented in this study are included in the article. Further inquiries can be directed to the corresponding authors.
